# Particled *Mica*, STB-HO has chemopreventive potential via G1 arrest, and inhibition of proliferation and vascular endothelial growth factor receptor 2 in HCT colorectal cancer cells

**DOI:** 10.1186/1472-6882-13-189

**Published:** 2013-07-24

**Authors:** Sung-Yun Cho, Hyo-Jeong Lee, Sun-Mi Cho, Bonglee Kim, Yeon Kwon Jung, Sung-Hoon Kim

**Affiliations:** 1 Cancer Preventive Material Development Research Center, College of Korean Medicine, Kyung Hee University, 1 Hoegi-dong, Dongdaemun-gu, 130-701, Seoul, South Korea; 2 SeoBong Biobestech Co, 831 Yeoksam-dong, Gangnam-gu, Ltd.Hye Cheon Bldg #401, 135-080, Seoul, Republic of Korea

**Keywords:** Mica, VEGFR, Colon cancer, G1 arrest, Cell proliferation

## Abstract

**Background:**

Though *Mica*, a thin and sheet like mineral, has been used as a mineral medicine for treatment of bleeding, dysentery and inflammation in traditional medicine including Ayurveda, the biological evidences of *Mica* were not clearly elucidated so far. Thus, in the present study, the antitumor mechanism of particled *Mica* (STB-HO) was examined in colorectal cancers.

**Methods:**

Athymic nude mice were inoculated with HCT116 colon cancer cells and orally administered STB-HO daily for 41 days, and HCT116 and human umbilical vein endothelial cells (HUVECs) were treated with STB-HO for 0 ~ 24 hours to perform immunoblotting, cytotoxicity assay, FACs analysis and measurement of matrix metalloproteinase 9 (MMP-9) secretion and other experiments. Significant differences of all date were evaluated using Student’s t-test and a Turkey-Kramer multiple-comparison post test.

**Results:**

STB-HO significantly suppressed the tumor volume and weight in athymic nude mice inoculated with HCT116 cells at a dose of 100 mg/kg. Thus, the *in vivo* antitumor mechanism of STB-HO was to elucidated *in vitro* as well. STB-HO exerted cytotoxicity in HCT116, SW620 and HCT15 colorectal cancer cells. Also, STB-HO increased G1 cell population in a time and concentration dependent manner, enhanced the expression of p21, p27, p53 as cyclin dependent kinase (CDK) inhibitors, attenuated the expression of proliferating cell nuclear antigen (PCNA) and cyclin D1 and also reduced the production of vascular endothelial growth factor (VEGF) and matrix metalloproteinase 9 (MMP-9) in HCT116 cells. Consistently, STB-HO suppressed the phosphorylation of VEGFR2 in HCT116, SW620 and HCT15 cells. Also, STB-HO inhibited the VEGF mediated proliferation and also attenuated the phosphorylation of VEGFR2 and Akt in human umbilical vein endothelial cells (HUVECs).

**Conclusions:**

Collectively, these findings suggest that STB-HO has chemopreventive potential via G1 arrest and inhibition of proliferation and VEGFR2 in HCT116 colorectal cancer cells.

## Background

Colorectal cancer (CRC) is one of the leading causes of mortality in the western world. Chemotherapy including 5-fluorouracil (5-FU) therapy and surgical resection are well known methods for colon cancer treatment [1]. However, the side effects are induced by chemotherapy such as oral mucositis, diarrhea, neuropathy, anemia and alopecia [2]. Thus, recently natural products [3] and compounds [4-6] were reported to have antitumor effects in colorectal cancers alone or in combination with anticancer agents [7] with low toxicity. Also, Hu *et al*. suggested that among men and women taking vitamin and mineral supplements for more than 5 years, significant inverse associations with colon cancer were most pronounced among women with distal colon cancer [8]. Similarly, there are evidences that mineral selenium has antitumor activity in colon cancers [9-12].

The *Mica* group of sheet silicate minerals are generally classified as trioctahedral Mica including Biotite, Lepidolite, Muscovite, Phlogopite, Zinnwaldite and interlayer deficient *Mica*[13]. *Mica* has been used for decoration and treatment for bleeding, dysentery and inflammation in traditional medicine including Ayurveda for ages. Nasrin *et al*. showed no toxicity of Chondrokola Rosh, a traditional metallic Ayurvedic preparation, including various roasted metals (Hg and Cu), non-metal (sulphur and *Mica*) and medicinal herbs [14]. Also *Mica* was known to protect gastric mucosa by improving blood flow and inflammatory response [15] as well as suppress gastric cancer via regulation of p16 and Bcl-2 in rats [16], indicating *Mica* can be used as a medicine [17]. Thus, in the present study, antitumor mechanism of particled *Mica* (STB-HO) was examined *in vitro* in HCT116 colorectal cancer and human umbilical vein endothelial cells (HUVECs) and athymic nude mice inoculated with HCT116 cells.

## Methods

### Chemicals and reagents

STB-HO (particled *Mica*; Korea Patent Registration; 10–0454200) was supplied from Seobong Biobestech Company (Seoul, Republic of Korea). SW620, HCT116 and HCT15 human colorectal adenocarcinoma cells from the American Type Culture Collection (ATCC, Manassas, VA, USA) were maintained in RPMI 1640 supplemented with fetal bovine serum (FBS), liquid gentamicin reagent solution, penicillin and streptomycin (PEST), and trypsin EDTA were purchased from Gibco (Carlsbad, CA, USA). Human umbilical vein endothelial cells (HUVECs) cells from the American Type Culture Collection (Manassas, VA, USA) were maintained in M199 supplemented with 20% fetal bovine serum (FBS), liquid gentamicin reagent solution, penicillin and streptomycin (PEST), 3 ng/ml bFGF, 5 units/ml heparin. Enhanced chemiluminescence (ECL) Western blotting detection reagents and Hyperfilm ECL were from Amersham-Pharmacia Korea (Seoul, Korea). Anti-rabbit IgG heavy and light chain-specific (rabbit, mouse) peroxidase conjugates and antibody against p21, p27, p53, pp53, cyclin D1, pAKT, AKT, PI3K and PCNA were purchased from Cell signaling technology (Denver, MA, USA). Antibodies of VEGFR2 and pVEGFR2 were purchased from Santa Cruz Biotechnology (Santa Cruz, CA, USA). β-actin was purchased from Sigma Chemical Co. (St. Louis, MO, USA). VEGF and MMP-9 ELISA kit were purchased from Invitrogen (Carlsbad, CA, USA). Human recombinant VEGF was purchased from R&D systems (Minneapolis, MN, USA). Cell Proliferation ELISA kit was purchased from ROCHE (F. Hoffmann-La Roche Ltd, Switzerland). All other reagents used were purchased from Sigma Chemical (St Louis, MO, USA).

### Cell culture

SW620(ATCC CCL-227™), HCT116(ATCC CCL-247™) and HCT15 cells(ATCC CCL-225™) were seeded onto 100 mm Falcon plates at 2 × 10^6^ cells/mL in RPMI 1640 supplemented with 10% FBS and 1% penicillin/streptomycin. The cells were cultured at 37°C in a humidified atmosphere containing 5% CO_2_ to 60–80% confluence and then used for Western blot analysis. STB-HO was treated to various human colon cancer cells for 24, 48, 72 and 96 h. HUVECs were maintained in M199 plus 20% heat-inactivated fetal bovine serum (FBS), 3 ng/ml bFGF, 5units/ml heparin, 100 units/ml antibiotic-antimycotic solution (complete M199) in 0.1% gelatin coated flasks and incubated at 37°C in a humidified atmosphere containing 5% CO_2_. Once confluent, the cells were detached by trypsin-EDTA solution and used in experiments from the third to the sixth passages.

### Cytotoxicity assay

Cytotoxicity of STB-HO was evaluated by 3-(4,5-dimethylthiazol-2-yl)-2,5-diphenyl tetrazolium bromide (MTT) assay. Briefly, HUVECs were seeded onto 0.1% gelatin coated 96-well microplates at a density of 5×10^3^ cells per well and treated with various concentrations of STB-HO (0, 15.63, 31.25, 62.5,125, 250, 500, or 1000 μg/ml) for 48 h. After indicated incubation times, MTT (1 mg/ml) (Sigma Chemical Co., St. Louis, MO) solution was added for 2 h and MTT lysis buffer (20% SDS and 50% dimethylformamide) was then added for overnight. Optical density (OD) was measured using a microplate reader (TECAN, Austria) at 570 nm. Cell viability was calculated as a percentage of viable cells in STB-HO treated group versus untreated control by following equation.

$$\begin{array}{ll} \text{Cell}\;\text{viability}\;\left(\%\right)= & \frac{\left[O.D.\left(\text{STB}-\mathrm{HO}\right)-O.D.\left(\text{Blank}\right)\right]}{\left[O.D.\left(\text{Control}\right)-O.D.\left(\text{Blank}\right)\right]}\\ & \times 100 \end{array}$$

### Proliferation assay

Cell proliferation in HCT116 cells with STB-HO was evaluated as described by using Cell proliferation ELISA kit (Roche, Swiss) according to the manufacturer’s instructions. Briefly, after 48 h treatment of STB-HO, the cells were added by 10 μl/well of bromodeoxyuridine (BrdU) solution and reincubated for 2 h at 37°C. Then, BrdU solution was removed and 200 μl of FixDenat was added to each well. After incubation for 30 min at room temperature, FixDenat solution was removed and 100 μl of anti-BrdU-POD working solution was added to each well. After washing with PBS three times, 100 μl of substrate solution was added to each well and the optical density was measured at 450 nm using microplate reader (Molecular Devices Co., Sunnyvale, CA, USA). All samples were prepared in triplicates and the assay was repeated at least three times.

### Cell cycle analysis

HCT116 cells were treated with STB-HO (250 and 500 μg/ml) for 24, 48 and 72 h. The cells were fixed in 75% ethanol at -20°C and treated with RNase A (10 mg/ml) for 1 h at 37°C, stained with propidium iodide (PI) (50 μg/ml) and analyzed for the DNA content by FACSCalibur (Becton–Dickinson, Franklin Lakes, NJ, USA) using CellQuest Software (BD Bio-sciences, San Jose, CA, USA).

### Western blotting

Cells (5×10^6^ cells) treated with STB-HO were lyzed by using lysis buffer (50 mM Tris–HCl, pH 7.4, 300 mM NaCl, 0.5% Triton X-100, 0.1% SDS, 5 mM EDTA, and protease inhibitor cocktail). The extracts were incubated on ice for 30 min, and then centrifuged at 13,000×g for 30 min at 4°C and the supernatants were collected for western blotting. Protein concentrations were determined by Bradford assay (Bio-Rad), and equal amounts of proteins (30 μg) were separated by electrophoresis sodium dodesyl sulfate polyacrylamide gel electrophoresis (SDS-PAGE) and transferred to PVDF membranes (Amersham Biosciences, Piscataway, NJ, USA). The membranes were blocked with 5% skim milk in Tris-buffered saline containing 0.1% Tween 20 for 2 h at room temperature. The membranes were probed overnight at 4°C with mouse anti-human β-actin (1:1000; Sigma Aldrich, St. Louis, MO, USA), anti-human pAKT, AKT, p21, p27, p53, pp53, cyclin D1, PCNA and PI3K (1:1000; Cell signaling, Danvers, MA, USA), anti-human VEGFR2 and pVEGFR2 (1:500; Santa Cruz Biotechnology, CA, USA) followed by washing and incubation with HRP conjugated secondary antibody (AbD Serotec, Raleigh, NC, USA). Immunoreactive bands were visualized using the ECL system (Amersham-Pharmacia, Seoul, Korea).

### Measurement of VEGF and MMP-9 production by ELISA

VEGF and MMP-9 levels in HCT116 cells treated with STB-HO were measured using VEGF and MMP-9 ELISA kit (Invitrogen, Carlsbad, CA, USA) according to the manufacturer’s instructions. Briefly, the culture supernatants was added onto a 96-well microplate, and incubated for 2 h at room temperature. The plate was then washed four times with washing buffer and 100 μl of biotin conjugate was placed to each well for 1 h at room temperature. After washing four times with washing buffer, 100 μl of the stabilized chromogen was placed to each well and incubated for 30 min at room temperature in dark. Finally, 100 μl of stop solution was added to each well and the optical density was measured at 450 nm using microplate reader (Molecular Devices Co., Sunnyvale, CA, USA).

### HCT116 xenograft model

Four-week-old female BALB/c athymic nude mice (18 ± 3 g) were purchased from Chung-Ang Laboratory Animals (Seoul, Korea) and housed in animal facility at 22 ± 3°C and 60 ± 10% humidity with light-controlled (12 h, 07:00–19:00) environment. All materials including bedding and feed were sterilely cleaned by UV rays for 30 min before treatment to the mice. The animal study was conducted under the guidelines approved by Institutional Animal Care and use Committee, Kyung Hee University [KHUASP(SE)-11-005] as previously described with minor modifications [18]. Briefly, 2 × 10 ^6^ of HCT116 cells were mixed with Matrigel (Becton Dickinson, 50% in 100 μl) and injected subcutaneously into the right flank of 6-week-old male BALB/c athymic nude mice (Chung-Ang Laboratory Animals, Seoul, Korea)) for 3 groups (Control and two STB-HO treated groups). After 1 week adaptation, the animals were assigned to four groups (n = 6): negative control (vehicle (saline) + HCT116 inoculation), STB-HO50 (50 mg/kg+ HCT116 inoculation), and STB-HO100 (100 mg/kg+ HCT116 inoculation). Everyday STB-HO dissolved in saline was orally treated to the athymic nude mice for 41 days during experiment period. Tumor size was monitored twice a week with a caliper, and tumor volume was also calculated as described [19]. At the end of animal study, tumors were dissected, weighed and photographed.

### Data analyses

Data were shown as means ± SE. Significant differences were evaluated using Student’s *t*-test and a Turkey-Kramer multiple-comparison post test.

## Results

### STB-HO suppresses tumor growth in HCT116 xenograft model

**Figure 1:**
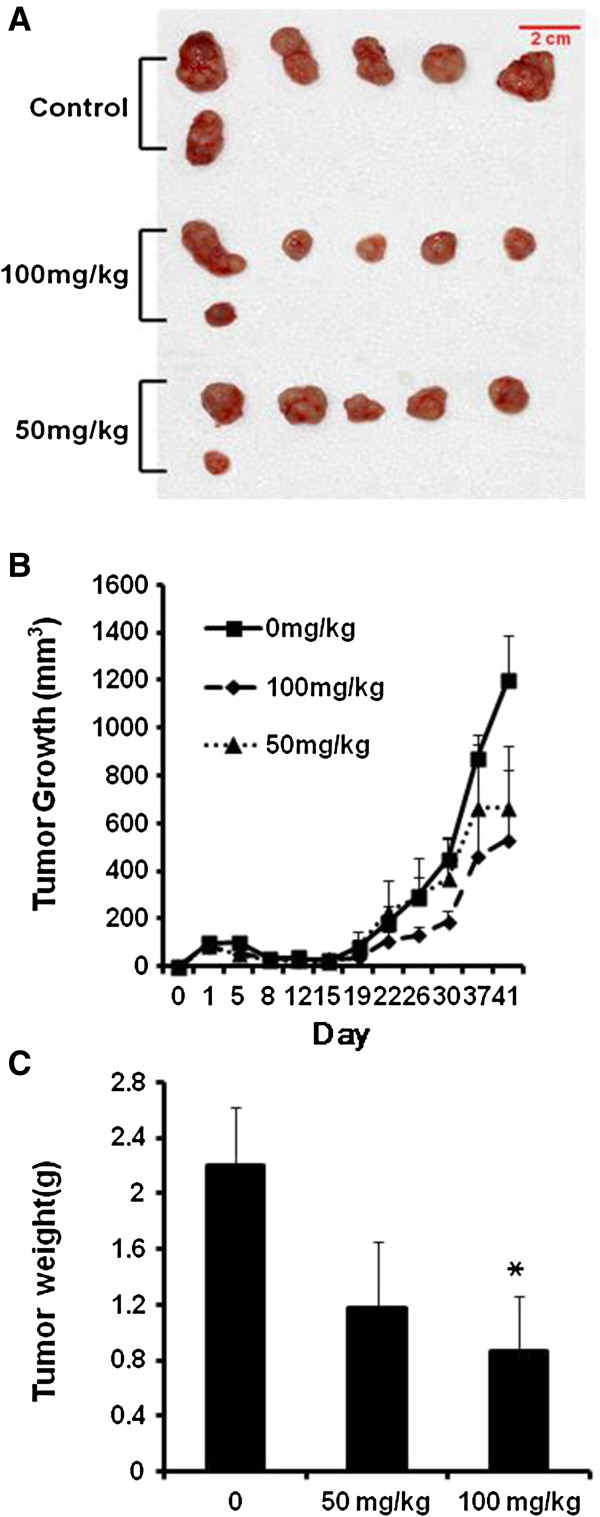
**Effect of STB-HO on the tumor weight and growth in athymic nude mice inoculated by HCT116 cells.** From three days after HCT116 cell inoculation, STB-HO (50 and 100 mg/kg body weight) was orally administered daily. **(A)** Photographs of dissected tumors in mice. **(B)** Tumor growth in a time course. **(C)** Final tumor weight at the termination of experiment. Values were means ±SD, n=6. *p < 0.05 and ***p<0.001 compared to untreated control.

As shown in Figure 1B, STB-HO suppressed the growth of HCT116 cancer cells inoculated in BALB/c athymic nude mice at the doses of 50 and 100 mg/kg without affecting body weight (Data not shown). Consistently, Treatment of STB-HO reduced the tumor weight in a dose dependent manner compared to untreated group following animal sacrifice, but statistical significance was recognized only between control and STB-HO (100 mg/kg) treated group (Figure 1A, C).

### STB-HO inhibits cell proliferation in human colorectal cancer cell lines

**Figure 2:**
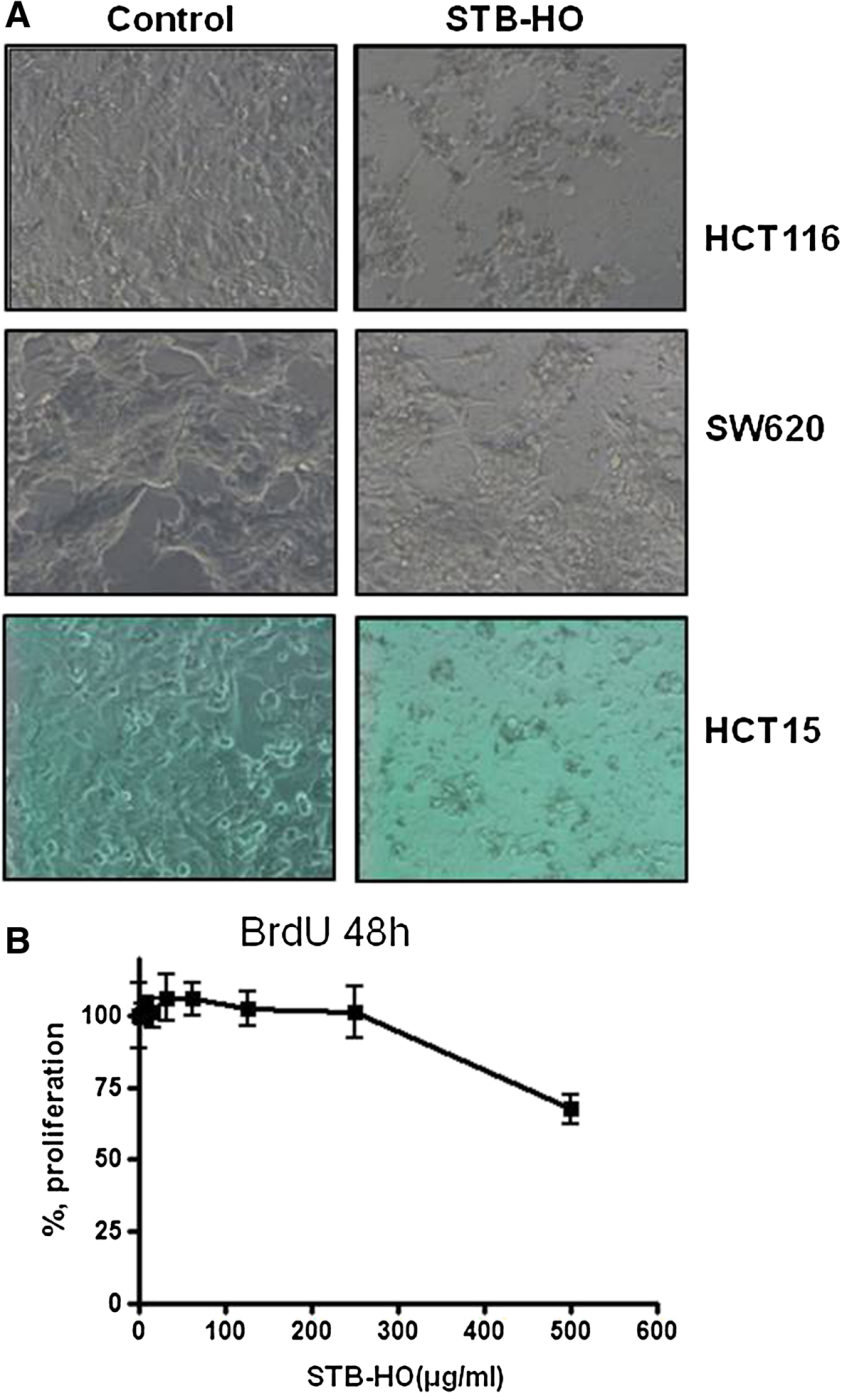
**Effect of STB-HO on the morphology of HCT116, SW620 and HCT15 cells and the proliferation of HCT116 cells. (A)** STB-HO was treated to HCT116, SW620 and HCT15 cells for 96 h, and its cell images were taken by using microscope (X200). **(B)** HCT116 cells were treated with STB-HO for 48 h and cell proliferation was measured by using BrdU proliferation ELISA kit (Roche, Swiss).

We first investigated whether STB-HO can suppress the proliferation of human colon cancer cell lines. After treatment with STB-HO in human colon cancer cell lines for 96 h, cell morphology was observed using microscope. As shown Figure 2A, STB-HO significantly suppressed cell proliferation in human colon cancer cells. Especially, the suppression of cell proliferation by STB-HO treatment was more effective in HCT 116 cancer cells compared to other colorectal cancer cells such as SW620 and HCT15 cells. Consistently, BrdU assay revealed, as shown in Figure 2B, the proliferation of HCT116 cells was decreased in a concentration dependent manner by STB-HO treatment, implying that STB-HO inhibits the proliferation of colorectal cancer cells.

### STB-HO induces G1 arrest in HCT116 colorectal cancer cells

**Figure 3:**
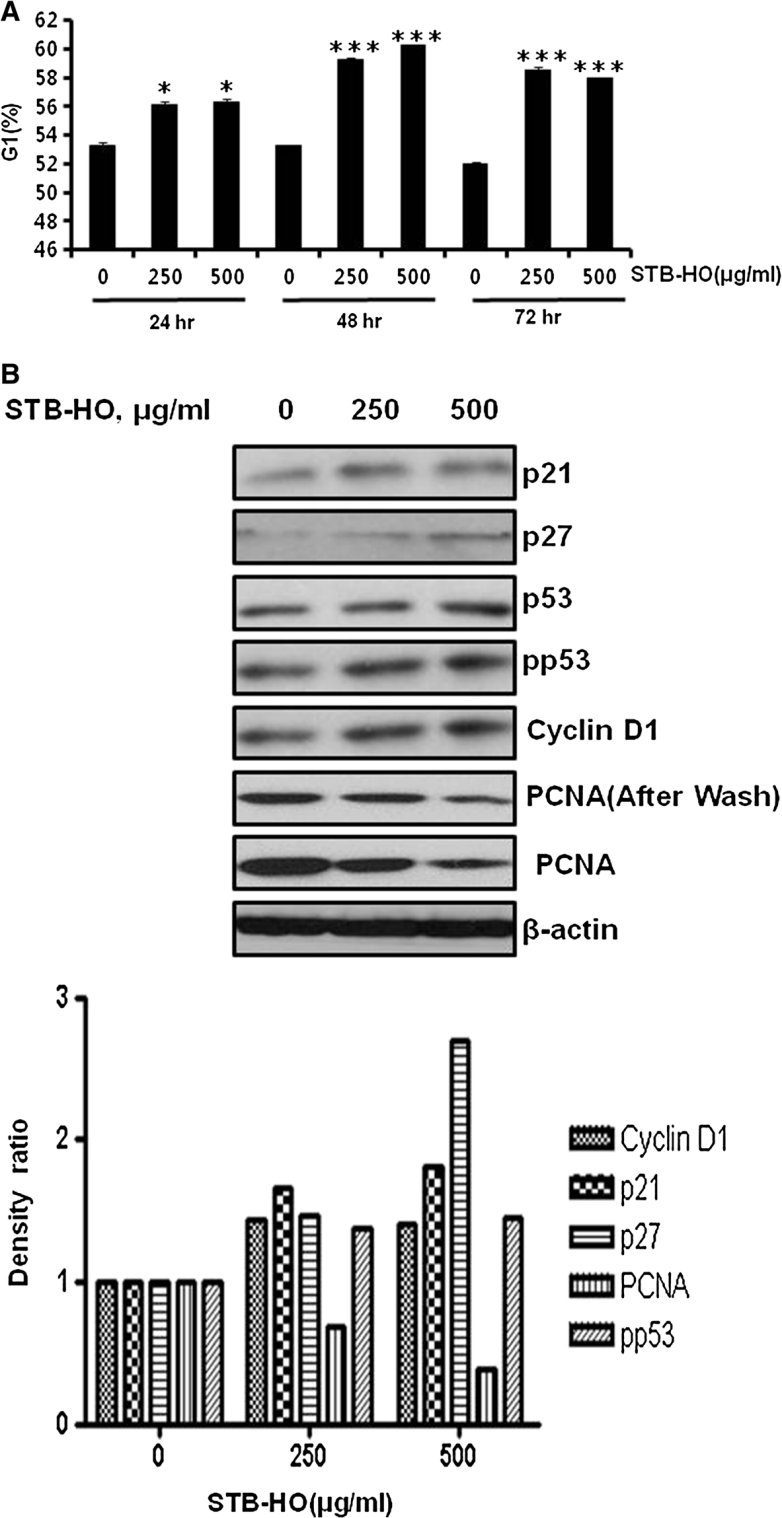
**Effect of STB-HO on G1 arrest and cell cycle related genes in HCT116 cells. (A)** Cells were treated with STB-HO (0, 250 or 500 μg/ml) for 24, 48 and 72 h. Cell cycle distribution was analyzed by flow cytometry. Bar graphs represent the percentage of the sub-G1 apoptotic DNA fraction. Data are presented as means ± S.D. *p < 0.05 and ***p < 0.001 compared between each time’s control and STB-HO (250 or 500 μg/ml) treated groups. **(B)** HCT116 cells were treated with or without STB-HO (0, 250 or 500 μg/ml) for 24 h. Cell lysates were prepared and subjected to Western blotting to determine the expression of p21, p27, p53, pp53, Cyclin D1, PCNA and β-actin. Band density of β-actin, p21, p27, pp53, PCNA and Cyclin D1 were quantified using Gelpro analyzer (Media Cybernetics, Bethesda, MD, USA).

Cell cycle analysis was performed to find out the effect of STB-HO in HCT116 cancer cells. STB-HO significantly increased G1 population in HCT116 cells in a time dependent manner (Figure 3A). One day after STB-HO treatment, the expression of p21, p27 and pp53 as CDK inhibitors was significantly increased in HCT116 cells (Figure 3B). In addition, STB-HO suppressed the expression of cyclin D1 and PCNA which are regulating cell cycle (Figure 3B). These data indicate that STB-HO induces G1 arrest which is crucial to inhibit proliferation and induce apoptosis in HCT116 colorectal cancer cells.

### STB-HO suppresses the production of VEGF and MMP-9 in HCT 116 colorectal cancer cells

**Figure 4:**
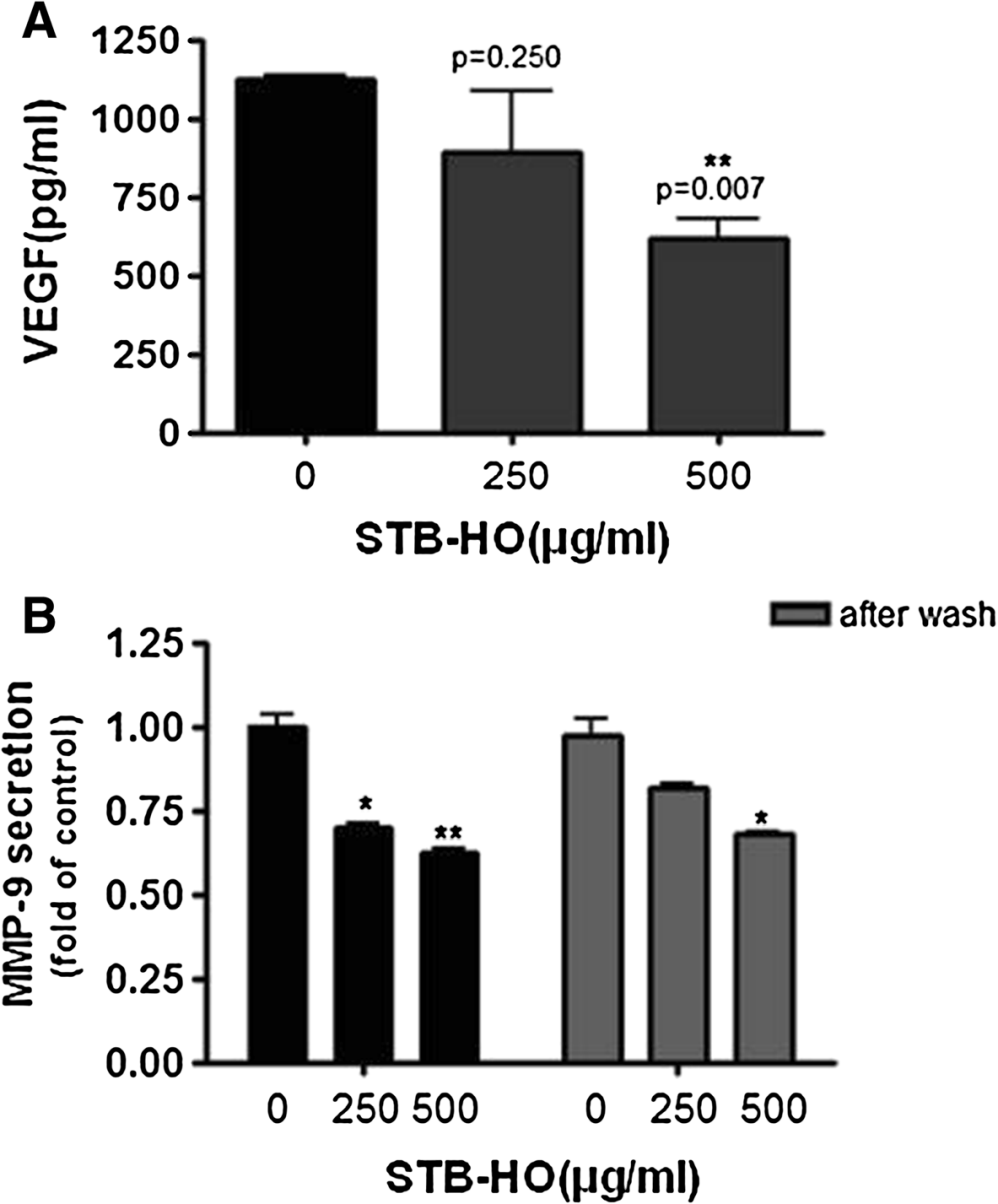
**Effect of STB-HO on VEGF production and MMP-9 secretion in HCT116 cells.** HCT116 Cells were treated with or without STB-HO (0, 250 or 500 μg/ml) for 48 h. **(A)** STB-HO inhibits vascular endothelial growth factor (VEGF) production. VEGF level was measured by ELISA. After removal of the supernatant, HCT116 cells were washed by PBS. The cells were cultured in completed RPMI media for 48 h. Then VEGF level was measured by ELISA. **(B)** STB-HO inhibits matrix metalloproteinase (MMP-9) production. MMP-9 level was measured by ELISA. Data are presented as means ± S.D. * *p* < 0.05, ** *p* < 0.01 and *** *p* < 0.001 compared to untreated control.

We also examined the effect of STB-HO on the production of VEGF and MMP-9 which are closely associated with metastasis and angiogenesis [20,21]. HCT 116 cancer cells were exposed to STB-HO for 48 h and, VEGF and MMP-9 levels were measured by ELISA. VEGF and MMP-9 production that are associated with angiogenesis and metastasis was significantly decreased in a dose dependent manner in HCT 116 colon cancer cells by STB-HO as shown in Figure 4A and Figure 4B. Also, though further changing medium one day later, the production of VEGF and MMP-9 was still suppressed in HCT 116 cancer cells, implying that STB-HO may exert anti-angiogenic activity in cancer cells.

### STB-HO suppresses VEGFR2 and PI3K/Akt signaling in colorectal cancer cells

**Figure 5:**
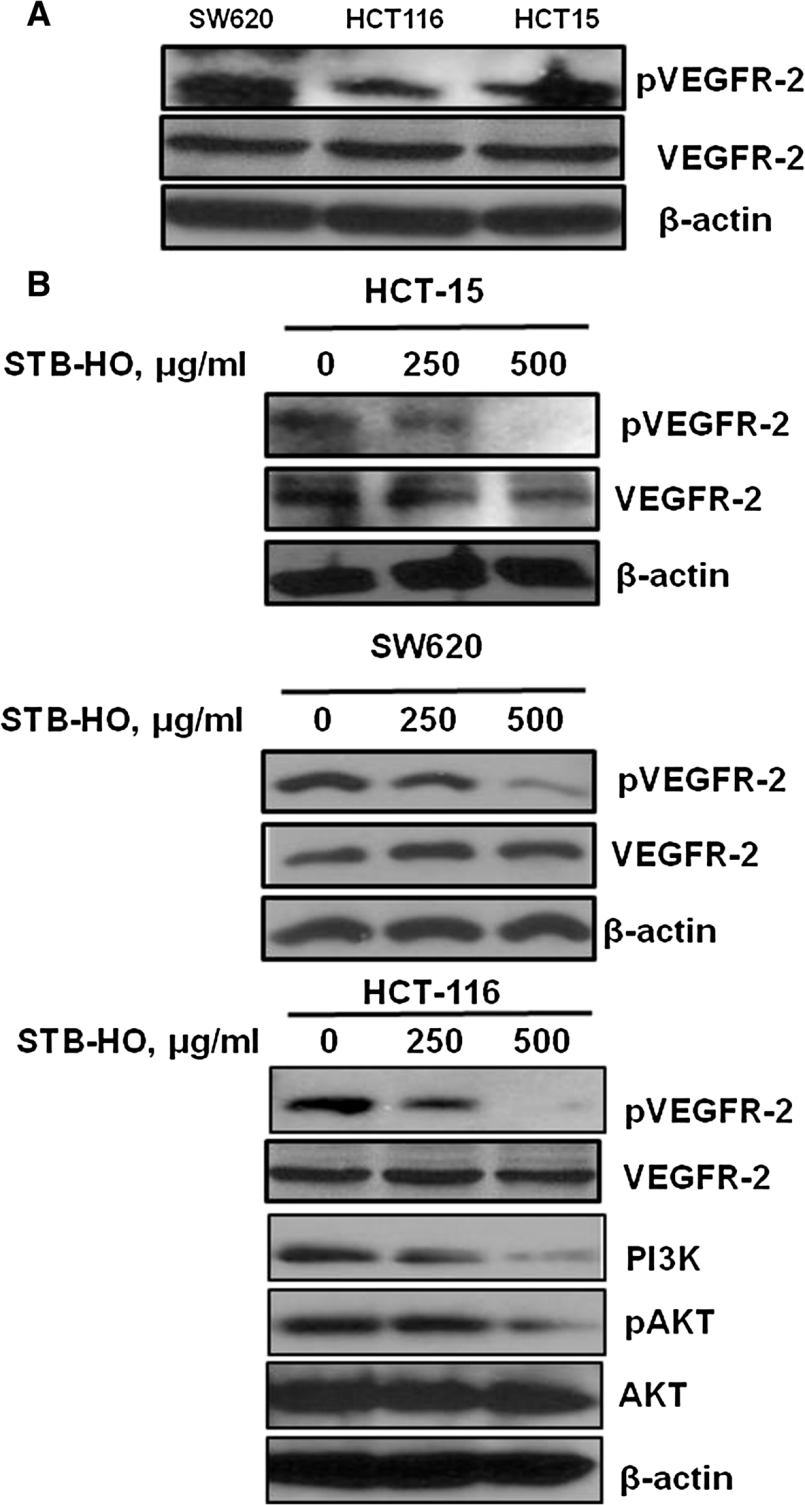
**Effect of STB-HO on pVEGFR2, PI3K and Akt in colon cancer cells. (A)** Basal expression of pVEGFR2 was confirmed in various colon cancer cells by Western blotting. **(B)** STB-HO (0, 250 or 500 μg/ml) was treated in HCT15, SW620 and HCT116 colon cancer cells for 24 h. Western blotting was performed to determine the expression of pVEGFR2, VEGFR2, PI3K, pAKT, AKT and β-actin in STB-HO treated colon cancer cells.

VEGF receptor (VEGFR) is crucial to promote tumor progression, angiogenesis and proliferation by binding to VEGF. The basal expression of VEGFR-2 was confirmed in colorectal cancer cells such as SW620, HCT116 and HCT15 (Figure 5A). We also found that the phosphorylation of pVEGFR2, PI3K and pAKT was attenuated in three colon cancer cells by STB-HO (Figure 5B), demonstrating STB-HO can abrogate the activity of proliferation in cancer cells via suppression of pVEGFR2, PI3K and pAKT.

### STB-HO inhibits VEGF mediated proliferation and phosphorylation of VEGFR2 and Akt in HUVECs

**Figure 6:**
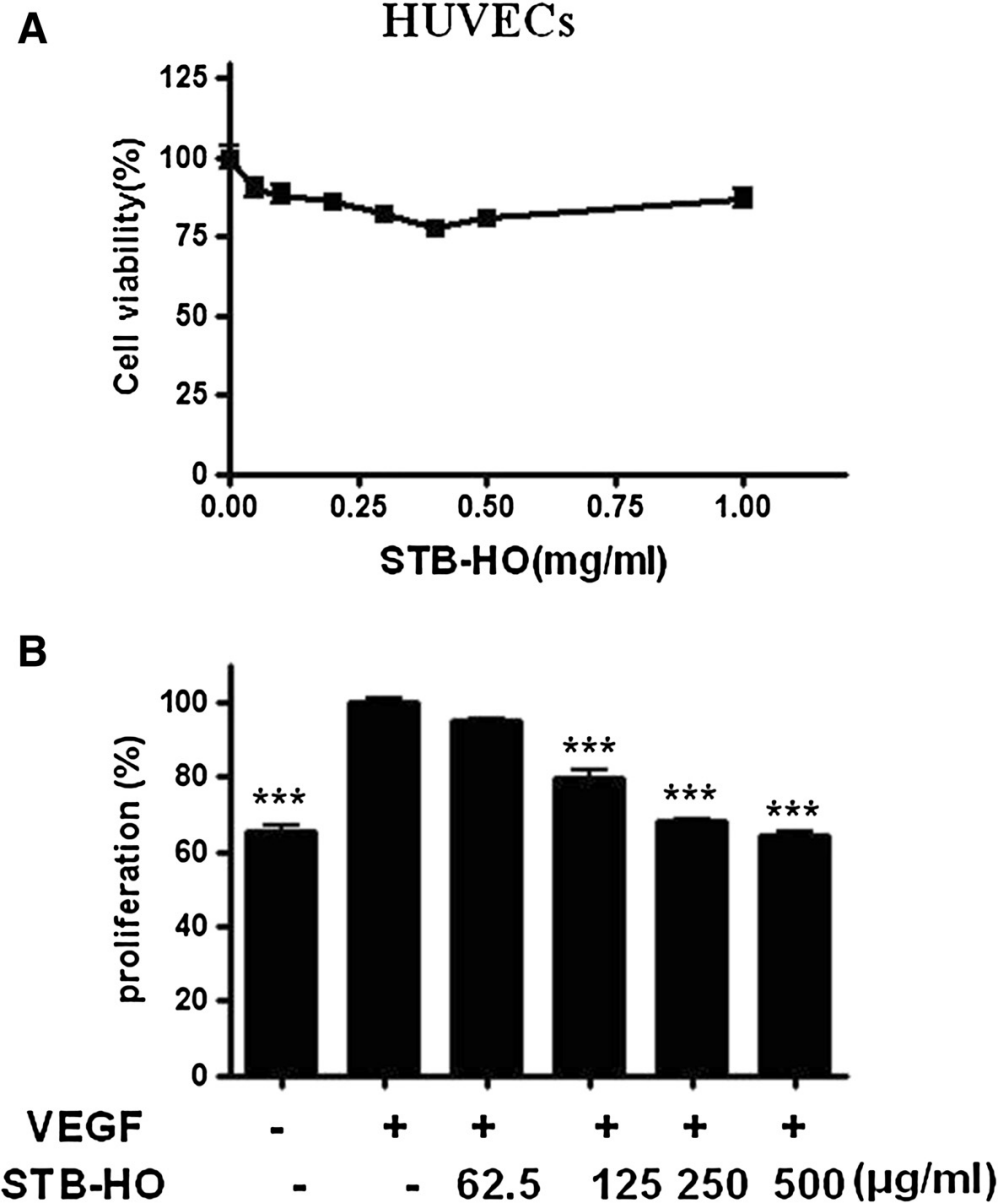
**Effect of STB-HO on the viability and VEGF mediated proliferation of HUVECs. (A)** Various concentrations of STB-HO were treated to HUVECs and cytotoxicity of STB-HO in HUVECs was measured by MTT assay. **(B)** HUVECs were exposed to STB-HO in M199 containing 1% heat-inactivated FBS, heparin (5 units/ml) and VEGF 10 ng/ml for 48 h, and the VEGF mediated proliferation was assessed by MTT assay. The absorbance was measured by ELISA reader. Data were presented as means ± S.D. * *p* < 0.05, ** *p* < 0.01 and *** *p* < 0.001 compared with VEGF treated control. All samples were prepared in triplicates and the assay was repeated at least three times.

**Figure 7:**
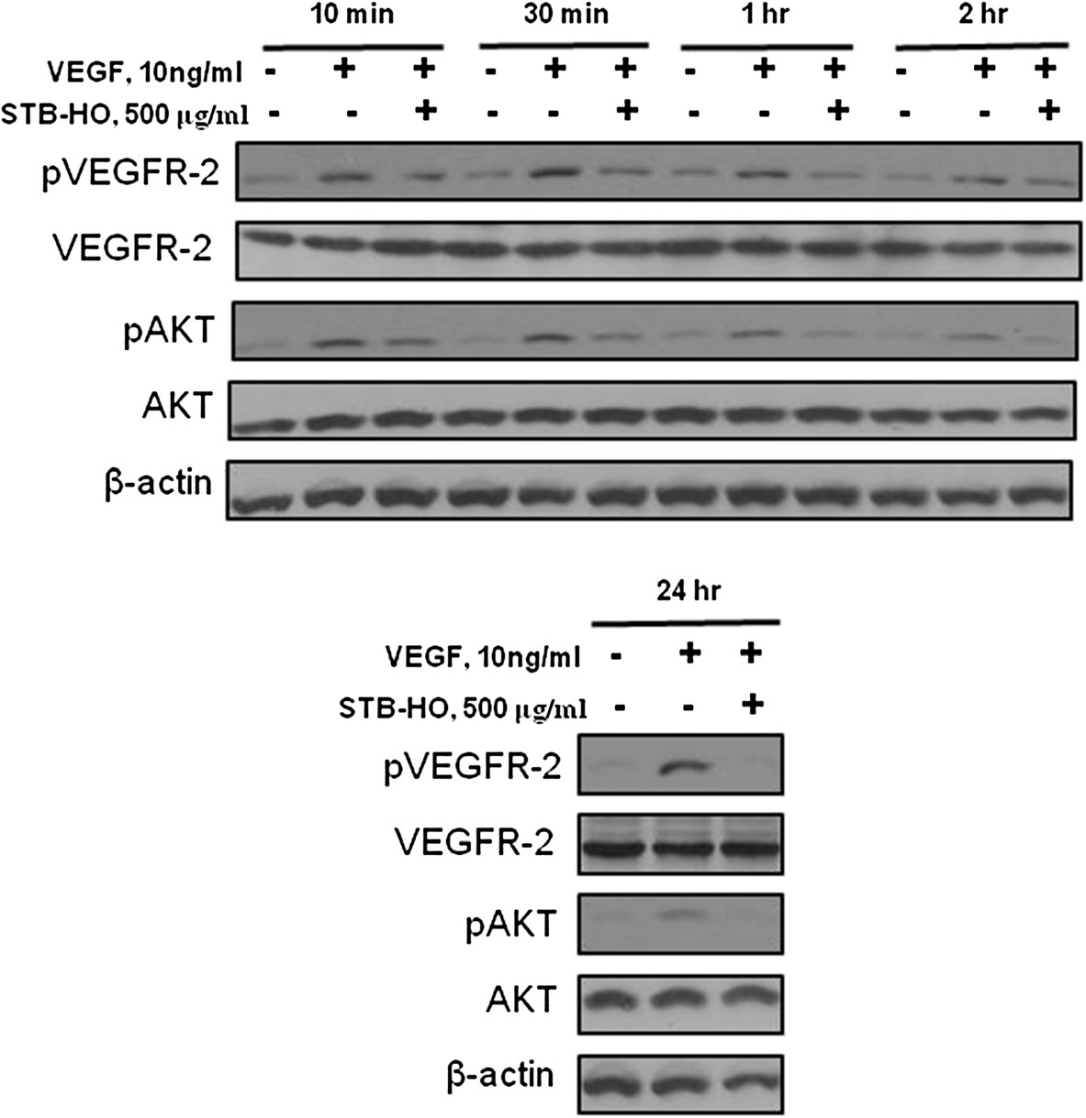
**Effect of STB-HO on pVEGFR2, PI3K and Akt in HUVECs.** HUVECs were starved for 24 h in M199 containing 1% heat-inactivated FBS and then treated with various concentrations of STB-HO (0, 250 or 500 μg/ml) in M199 containing 1% heat-inactivated FBS, 10 ng/ml VEGF and 5 units/ml heparin for 10 min, 30 min, 1 h, 2 h, and 24 h. The cells were harvested and western blotting was performed to determine the expression of pVEGFR2, VEGFR2, pAKT, AKT and β-actin.

As shown in Figure 6A, MTT assay revealed that STB-HO did not show any cytotoxicity in HUVECs as a normal cell line. Also, to confirm antiangiogenic activity of STB-HO in HUVECs, proliferation assay was performed in VEFG treated HUVECs by MTT assay. As shown in Figure 6B, STB-HO inhibited VEGF-induced proliferation of HUVECs in a dose dependent manner at nontoxic concentrations in HUVECs. In addition, as shown in Figure 7, STB-HO suppressed the phosphorylation of VEGFR-2 and Akt in HUVECs compared to untreated control.

## Discussion

There are evidences that minerals have antitumor activity in several cancers. For instances, arsenic trioxide (As_2_O_3_) was known to treat breast cancer [22] and colon cancer cells [23,24], selenium (Se) was reported to have antitumor potential in several cancers such as colon [25,26], prostate [27,28], zinc (Zn) was reported to have potential therapeutic for chemoresistant ovarian cancer [29] and also cadmium (Cd) induced mitogenic signaling in breast cancer cell by an ER alpha-dependent mechanism [30]. Similarly, in the present study, mineral *Mica* (STB-HO) showed antitumor potential in colorectal cancers. Though STB-HO exerted anti-proliferative activity in HCT116, SW620 and HCT15 colorectal cancer cells, HCT116 cells are were more susceptible to STB-HO compared to two other colon cancer cells, since they are positive for transforming growth factor beta 1 (TGF beta 1) and beta 2 (TGF beta 2) expression with a mutation in codon 13 of the ras protooncogene [31].

Also, STB-HO increased G1 cell population in a time and concentration dependent manner and enhanced the expression of p21, p27, p53 as cyclin dependent kinase (CDK) inhibitors [32-34], attenuated the expression of proliferating cell nuclear antigen (PCNA) and cyclin D1, implying G1 arrest leading to cell death by STB-HO in HCT116 cells. Furthermore, STB-HO attenuated the expression of survival gene PCNA and reduced typical angiogenesis marker VEGF production in HCT116 cells, indicating anti-proliferative and anti-angiogenic activity of STB-HO in HCT116 cells.

VEGF is an important signaling protein involved in both vasculogenesis and angiogenesis. As an essential receptor protein tyrosine kinase propagating cellular signal transduction processes, VEGFR-2 is a central target for drug discovery against tumor-associated angiogenesis [35]. Consistently, STB-HO suppressed the phosphorylation of VEGFR2 in HCT116, SW620 and HCT15 cells and also inhibited the VEGF mediated proliferation as well as attenuated the phosphorylation of VEGFR2 and Akt in human umbilical vein endothelial cells (HUVECs), strongly demonstrating anti-angiogenic activity via inhibition of VEGFR2 signaling. Consistently, ELISA revealed that STB-HO reduced the production of VEGF and MMP-9 in HCT116 cells. Nevertheless, it was noteworthy that STB-HO suppressed the tumor volume and weight in athymic nude mice inoculated with HCT116 cells at a dose of 50 and 100 mg/kg through two animal studies. However, the *in vitro* effective concentration was high because of poor solubility of STB-HO in cell culture study, which should be improved by nanoparticle method, synthesis or new dilution methods in the near future.

## Conclusions

Mineral *Mica* (STB-HO) showed cytotoxicity in colorectal cancer cells, increased G1 arrest and, reduced VEGF production in HCT116 colorectal cancer cells, attenuated the phosphorylation of VEGFR2 and Akt in HUVECs and suppressed the tumor volume and weight in athymic nude mice inoculated with HCT116 cells. Collectively, these findings suggest that STB-HO has chemoprevntive potential via G1 arrest and inhibition of proliferation and VEGFR2 in HCT116 colorectal cancer cells.

## Abbreviations

HUVECs: Human umbilical vein endothelial cells; MMP-9: Matrix metalloproteinase 9; CDK: Cyclin dependent kinase; PCNA: Proliferating cell nuclear antigen; VEGF: Vascular endothelial growth factor; MTT: 3-(4,5-dimethylthiazol-2-yl)-2,5-diphenyl tetrazolium bromide; BrdU: Bromodeoxyuridine; VEGFR: VEGF receptor; ELISA: Enzyme-linked immunosorbent assay enzyme-linked immunospecific assay.

## Competing interests

The authors declare that they have no competing interests.

## Authors’ contributions

SYC carried out the study and wrote the manuscript. HJL carried out the study, designed the experiments and helped conceive the study. SMC and BLK carried out the study and contributed animal model. YKJ contributed reagents and materials. SHK conceived and designed experiments and revised the manuscript. All authors read and approved the final manuscript.

## Pre-publication history

The pre-publication history for this paper can be accessed here:

http://www.biomedcentral.com/1472-6882/13/189/prepub
